# Insights Into Cryoconite Community Dynamics on the Alpine Glacier Throughout the Ablation Season

**DOI:** 10.1002/ece3.71064

**Published:** 2025-03-24

**Authors:** Tereza Novotná Jaroměřská, Roberto Ambrosini, Dorota Richter, Miroslawa Pietryka, Przemyslaw Niedzielski, Juliana Souza‐Kasprzyk, Piotr Klimaszyk, Andrea Franzetti, Francesca Pittino, Lenka Vondrovicová, Antonella Senese, Krzysztof Zawierucha

**Affiliations:** ^1^ Department of Ecology, Faculty of Science Charles University Prague Czech Republic; ^2^ Department of Environmental Science and Policy University of Milan Milan Italy; ^3^ Department of Botany and Plant Ecology Wrocław University of Environmental and Life Science Wrocław Poland; ^4^ Department of Analytical Chemistry, Faculty of Chemistry Adam Mickiewicz University Poznań Poland; ^5^ Department of Water Protection, Faculty of Biology Adam Mickiewicz University Poznań Poland; ^6^ Department of Earth and Environmental Sciences University of Milano‐Bicocca Milan Italy; ^7^ Institute of Geochemistry, Mineralogy and Mineral Resources, Faculty of Science Charles University Prague Czech Republic; ^8^ Department of Animal Taxonomy and Ecology, Faculty of Biology Adam Mickiewicz University Poznań Poland

**Keywords:** Forni glacier, phenology, stable isotopes, supraglacial habitats, Tardigrada, top‐down control

## Abstract

Cryoconite holes (water reservoirs) significantly contribute to biodiversity and biogeochemical processes on glacier surfaces. However, the lack of seasonal observations of cryoconite biota limits our knowledge of glacial ecosystem functioning. We studied photoautotrophs, consumers and sediment characteristics (community structure, biomass, elemental composition, organic matter content, δ^13^C, δ^15^N) from cryoconite holes in the upper and lower parts of the Forni Glacier ablation zone (Italy) throughout the ablation season. Dominant cyanobacteria were Oscillatoriaceae and Leptolyngbyaceae, while dominant green algae were Zygnemataceae and Chlorellaceae. Tardigrades (*Cryobiotus klebelsbergi*) were the dominant consumers. The biomass of consumers negatively correlated with the biomass of green algae, indicating that grazing likely controls algal communities in the upper part. Green algae dominated the upper part, while a shift from green algae‐ to cyanobacteria‐dominated communities was observed in the lower part during the season. The increase in δ^13^C of cryoconite organic matter (OM) in the lower part followed the trend of the community shift of photoautotrophs potentially affected by precipitation. Also, δ^13^C of tardigrades positively correlated with δ^13^C of cryoconite OM in the upper part, indicating some cryoconite OM as their food. Some photoautotrophic taxa appeared only on specific dates, and no spatio‐temporal changes in the cryoconite general elemental composition were found. Our data indicate that changes in the community structure and biomass of cryoconite biota on the Forni Glacier likely depend on the interplay between phenology, stochastic events (e.g., rainfall) and top‐down or bottom‐up controls. We demonstrate that multiple observations are essential for understanding the ecology of biota inhabiting cryoconite holes throughout the ablation season.

## Graphical abstract

The biomass of consumers, tardigrades, negatively correlated with the biomass of green algae, indicating that grazing likely controls algal communities on this alpine glacier.

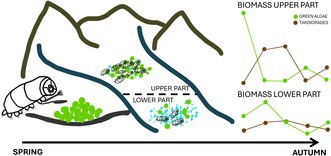

## 1 Introduction

Glaciers and ice sheets are one of the fastest‐changing biomes on Earth (Zemp et al. 2006; Bosson et al. 2023). Biological activity on the glacier surface (supraglacial environment) can affect the surface albedo (reflection of solar radiation) with potential implications for glacier melt dynamics (e.g., Stibal et al. 2012a; Yallop et al. 2012; Di Mauro et al. 2020). Thus, the knowledge of the drivers that influence the abundance, diversity and phenology of glacial biota is crucial for modelling how climate change may alter glacial ecosystems (Stibal et al. 2012a; Gobbi et al. 2021; Hodson et al. 2021).

Most biological processes within the supraglacial environment occur in the ablation zone (the area where the ice mass loss exceeds its increase) during the summer season (Cameron et al. 2012; Stibal et al. 2012a). At that time, glacier surfaces provide liquid water and suitable conditions for the activity myriad of organisms, from bacteria to invertebrates (e.g., Cameron et al. 2012; Zawierucha et al. 2018, 2021). On glacier surfaces, the highest biodiversity is found inside cryoconite holes, which are small water‐filled depressions in the glacial ice formed by cryoconite, a dark sediment that lowers the albedo of the glacier surface (Wharton et al. 1985; Takeuchi et al. 2001; Cameron et al. 2012; Rozwalak et al. 2022). Due to their pond‐like structure, cryoconite holes, and specifically the cryoconite, harbour a unique community of organisms, from microbes to minute invertebrates (Franzetti et al. 2017; Zawierucha et al. 2021; Poniecka et al. 2020). Microinvertebrates, in particular, are important supraglacial consumers in cryoconite holes, likely able to influence the community structure of microbes (Vonnahme et al. 2016), though their role in supraglacial ecology and trophic networks remains unclear (Novotná Jaroměřská et al. 2021; Zawierucha et al. 2018; Zawierucha et al. 2022).

Changes in the community structure of cryoconite holes over the ablation season have been minimally investigated (e.g., Takeuchi 2013; Musilova et al. 2015; Pittino et al. 2018; Winkel et al. 2022; Sanyal et al. 2024), and some studies involving seasonality present contrasting results. For example, Pittino et al. (2018) and Sanyal et al. (2024) showed that the supraglacial microbial community structure in the Alps and Antarctica changed over the ablation season, while Musilova et al. (2015) showed that the community structure appeared to be stable over the season on an Arctic glacier. Moreover, studies considering the biomass of different taxa, critical for supraglacial biogeochemical processes affecting albedo and, therefore, the mass balance of glaciers, from cryoconite holes are scarce and do not include seasonal observations (e.g., Buda et al. 2020).

The lack of data for both photoautotrophs and consumers throughout the ablation season in alpine cryoconite prevents the estimation and understanding of (i) ecological and trophic links of biota in cryoconite holes, which are difficult to resolve in ‘snapshot’ studies (e.g., Zawierucha et al. 2018), (ii) biological diversity on glaciers, since some taxa may appear only in a particular period during the ablation season (Pittino et al. 2018) and (iii) sudden community shifts, such as photoautotroph ‘blooms’ in cryoconite, which may result in punctuated increases in biological activity as well as supraglacial melt (Williamson et al. 2020).

In this study, we investigated the biomass and the community structure of photoautotrophs and their consumers, the stable isotopic composition (δ^13^C and δ^15^N) of consumers and OM in cryoconite and the general elemental composition of cryoconite on the Forni Glacier, one of the most extensively studied glaciers in the Alps (e.g., Citterio et al. 2007; Azzoni et al. 2016; Senese et al. 2020; Crosta et al. 2025). We aimed to approach how the supraglacial ecosystem changes over the ablation season.

## 2 Material and Methods

### 2.1 Study Site and Sampling

**FIGURE 1:**
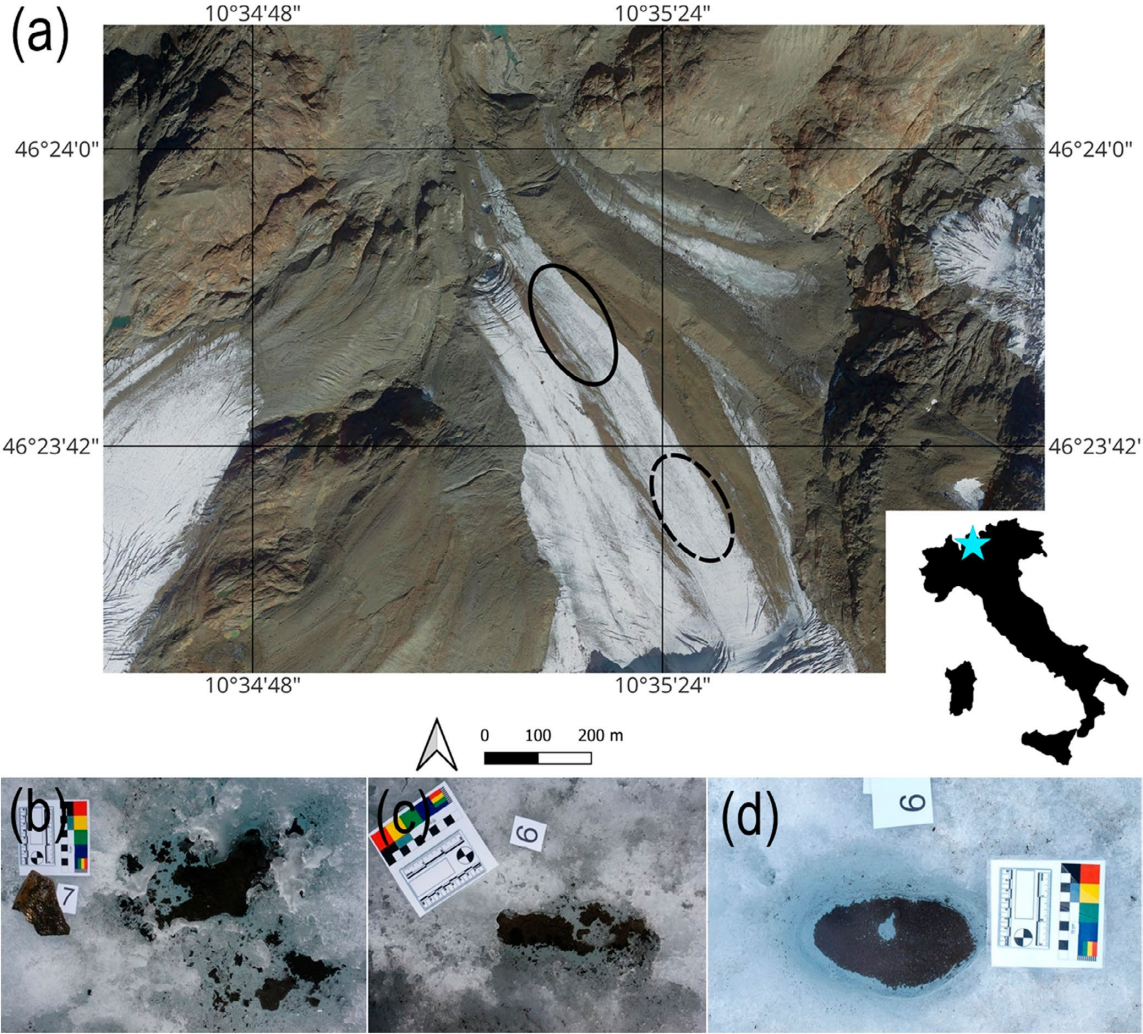
(a) Location of sampling areas on the Forni Glacier. The solid ellipse indicates the lower sampling area and the dotted ellipse indicates the upper sampling area. (b–c) cryoconite holes common in the lower part. (d) a cryoconite hole typical from the upper part of the ablation zone.

The Forni Glacier (Figure 1) is a valley‐type mountain glacier located in the Ortles–Cevedale group (Stelvio National Park, Italy) with an area of about 10.5 km^2^. The elevation of Forni Glacier ranges between 2600 and 3670 m a.s.l. (Senese et al. 2018). Cryoconite holes are located in the ablation zone at the glacier tongue. The ablation zone can be divided into two distinct parts. The lower part of the ablation zone is characterised by a mild slope, large amounts of debris including boulders, a narrower distance between moraines and closer proximity to the forefield. The upper part is characterised by a relatively flat surface, proximity to an ice fall, a lower amount of rocks and boulders on the ice surface and a wider distance between moraines.

Cryoconite samples were collected from the lower (approx. 2650 m a.s.l.) and the upper part (approx. 2700 m a.s.l.) of the ablation zone below the ice fall that connects the ablation and the accumulation area of the glacier. The horizontal distance between sampling areas was 250–300 m (Figure 1). Cryoconite was collected from the bottom of cryoconite holes during the 2019 ablation season on July 4 and 26, August 15 and 30 and September 19. Samples were collected to plastic test tubes with an aseptic stainless‐steel spoon. At each sampling date, cryoconite was collected from at least five holes to create one pooled sample at each part of the ablation zone. Up to 10 mL of cryoconite from the bottom of each hole was collected and frozen until further processing. Thereafter, material from each pooled sample was homogenised and split for subsequent analyses (see details below). For characterising meteorological conditions, we considered subhourly data of air temperature (Tsc) and liquid precipitation (Psc) acquired by a private weather station located at Santa Caterina Valfurva (1730 m a.s.l.) from June 1 to September 30 (for details, see Supporting Information 1). The modelled values of the temperature and precipitation on glacier along the sampling season were as follows: mean daily air temperature of +8.7°C, ranging from −6.1°C to +19.2°C, and mean daily precipitation of 16.6 mm, ranging from 0.8 to 82.8 mm (Figure S1).

### 2.2 Identification and Quantification of Photoautotrophs

Morphological observations were conducted using a Nikon Eclipse TE2000‐S digital microscope equipped with a Nikon DS‐Fi1 camera under 100× magnification. Literature for morphometric analysis and identification of photoautotrophs is provided in Supporting Information 1. Cyanobacteria, green algae and diatoms were identified to the lowest taxonomic level possible (generally genus), and the taxonomy and nomenclature of these groups were confirmed based on Algaebase (https://www.algaebase.org/).

For quantitative analyses, 20 μL of homogenised sample was placed on a glass slide under a coverslip in 10 repetitions. We measured 50 specimens from each taxon. The mean cell or filament size was then calculated considering the similarity of their geometric shapes. The resulting data (i.e., mean volume and number of cells in a given volume) were subsequently used to calculate the biomass of individual taxa. Algal biomass was calculated by assuming that the algal cell density is 1.0 g/cm^3^; therefore, the algal biomass is equal to its volume (Huot et al. 2007; Hutorowicz 2013).

### 2.3 Identification and Quantification of Top Consumers

Six millilitres of cryoconite was analysed after slow thawing at 3°C to avoid heat shock for consumers. Tardigrades were identified according to Mihelcic (1959), Dastych et al. (2003) and Zawierucha et al. (2019b). We extracted consumers from cryoconite on a constantly cooled Petri dish using a stereomicroscope (Olympus BZ51). All tardigrades were extracted with small shovels and counted. The density of animals was calculated per 1 cm^3^ and per 1 g of dry cryoconite.

### 2.4 Biomass of Tardigrades

The body length and width of individuals were measured on photographs taken by the Quick PHOTO Camera 3.0 software (Promicra, Prague, Czech Republic) under an Olympus BX53. Animals not suited for measurements (e.g., broken or bent) were not measured. The mass (wet mass, WM) of each specimen was calculated based on the formula of Hallas and Yeates (1972): if body length (L) and width (D) were 4:1, WM = L^3^ × 0.051 × 10^−6^, or 5:1, WM = L^3^ × 0.033 × 10^−6^.

### 2.5 Organic Matter in Cryoconite

The amount of organic matter was measured as a percentage of cryoconite weight loss. Air‐dried cryoconite samples were sieved through a 2‐mm mesh sieve to remove larger mineral fractions. No residues were found in any of the samples. The crucibles were washed with deionised water and placed in a thermostat at 50°C for 2 h to remove any residual moisture. Subsequently, the crucibles were transferred to a desiccator and weighed to the nearest 100 μg. Cryoconite samples were transferred to crucibles, placed for 24 h at 50°C, then transferred to a desiccator and weighed. Crucibles with cryoconite were then transferred to a muffle furnace and burned at 550°C for 3 h. After burning, when the temperature in the furnace was about 50°C, the crucibles were transferred to a desiccator and weighed to estimate the weight of the residual material.

### 2.6 General Elemental Composition of Cryoconite

The concentration of elements in combusted samples of cryoconite (without OM) was determined by a Plasma Quant MS (Analytik Jena, Germany) inductively coupled plasma mass spectrometry. All details on the procedure of preparation of material, calibration of instruments and validation of data are provided in Supporting Information 1. Only elements with more than 1000 μg/kg (Ca, K, P, Si, Al, Mg) were considered for further investigation.

### 2.7 Analyses of Carbon and Nitrogen Stable Isotopes

Tardigrades from slowly thawed cryoconite were prepared for the analysis according to Novotná Jaroměřská et al. (2021) and stored at −20°C. Before the analysis, all individuals for each sample were pooled, freeze‐dried and weighed into tin capsules.

For analyses of cryoconite, all animals (tardigrades, rotifers) were removed prior to further processing. Then, cryoconite was homogenised and oven‐dried at 45°C.

Cryoconite samples were divided into two groups: (i) for δ^15^N analysis, weighed and transferred to tin capsules without any further preparation; (ii) for δ^13^C analysis, carbonates were dissolved using 200 μL of 10% HCl moistened with diH_2_O with additions of 15, 15, 20, 50 and 100 μL, and drying after each addition equal to or up to 50 μL according to Brodie et al. (2011) with the modification after Vindušková et al. (2019). After the last acid addition, samples were left to dry at 50°C for 19 h. After drying, silver capsules were inserted into tin capsules and kept drying in a desiccator for at least 10 days. All cryoconite samples were prepared in three replicates. The analyses of δ^13^C and δ^15^N were performed using a Flash 2000 elemental analyser, Delta V Advantage isotope‐ratio mass spectrometer and a Continuous Flow IV system (all Thermo Fisher Scientific, Bremen, Germany) as described in detail by Novotná Jaroměřská et al. (2021). The stable isotope values were expressed in standard delta notation (δ) relative to the Pee Dee Belemnite for carbon isotopes and atmospheric N_2_ for nitrogen isotopes and normalised based on international standards.

### 2.8 Statistical Analyses

While examining variations in the biomass of photoautotrophs along the ablation season, the biomass was expressed as the relative biomass (e.g., ratio between the biomass of a taxon and the total biomass of all photoautotrophs). These ratios were log‐transformed and entered as dependent variables in linear regression models that included as predictors a continuous variable indicating the sampling date, expressed as progressive days since 1 July = 1 (e.g., August 15th = 46) and a dichotomous factor indicating the sampling area (i.e., either the lower or the upper part of the ablation zone). Additionally, we hypothesised that an interaction between these predictors may exist because the temporal trends may differ between the two study areas. We used a linear regression model also to test for δ^13^C, δ^15^N and OM content of cryoconite according to the same predictors. In all cases, we visually assessed model fits by plotting the residuals against the fitted values and by means of a qq‐plot, which showed some minor deviations from the model assumptions of normality of residuals and homogeneity of variance. To properly account for these problems, we moved to a permutation approach to assess the statistical significances. Indeed, permutation methods can provide exact control of false positives making only weak assumptions about the data (Winkler et al. 2014). To fit these models, we used the lmperm procedure in the permuco package version 1.1.0 (Frossard and Renaud 2019) of R version 3.5.3 (R Development Core Team 2018). Since the biomasses of photoautotrophic taxa were expressed as relative abundances, the different tests were non‐independent to one another. To keep the probability of detecting false positives to the nominal α = 0.05 across multiple tests, we first noted the *p*‐values of each of the linear models of photoautotrophs relative abundance, and then corrected them using the false discovery rate (FDR) procedure.

The biomass of tardigrades was assessed as the ratio between the sum of the biomasses of each specimen (see above) and the dry weight of the cryoconite from which they were extracted. These values were then log‐transformed to improve the fit of the models. We used a t‐test to investigate the variation of tardigrade biomass between the upper and the lower part of the ablation zone. Then, we investigated whether the biomass of tardigrades was related to that of photoautotrophs by using Pearson's correlations because we assumed potential mutual effects between consumers and photoautotrophs.

To compare δ^13^C and δ^15^N values between cryoconite and tardigrades, we used t‐tests while we used correlations to test for covariations between their values in the same cryoconite holes.

Finally, we tested whether the elemental composition (Ca, K, P, Si, Al, Mg) of cryoconite varied along the season with potentially different patterns in the two sampling areas. To this end, we used a redundancy analysis (RDA) where we entered as predictors the date (continuous covariate), the sampling area (dichothomous factor) and their interaction. The abundance of each element was standardised before the analysis. Significance of the RDA model was assessed through a permutation approach, and the analysis was conducted using the packages BiodiversityR version 2.13–1 (Kindt and Coe 2005) and vegan version 2.5–7 (Oksanen 2020).

## 3 Results

### 3.1 Photoautotrophs in Cryoconite Throughout the Ablation Season

The most abundant families of cyanobacteria were Oscillatoriaceae (*Phormidium* sp.) and Leptolyngbyaceae (*Leptolyngbya* sp.), while diatoms were represented by Stephanodiscaceae (*Cyclotella* sp.), Aulacoseiraceae ( *Aulacoseira granulata* Simonsen) and Bacillariaceae (*Nitzchia* sp.). The most abundant green algae families were Zygnemataceae (*Cylindrocystis brebisonii* f. *cryophila* Kol), Chlorellaceae (*Chlorella* sp.) and the genus *Trochiscia* (Figure S2, Table 1, Table S1). Other genera of cyanobacteria, green algae and diatoms were present only on some sampling dates.

**TABLE 1:** Presence (dark colour; at least one specimen in the sample) and absence (light colour) of specific cyanobacteria, green algae and diatom taxa in samples from the lower and upper part of the ablation zone at the Forni Glacier over the 2019 ablation season.

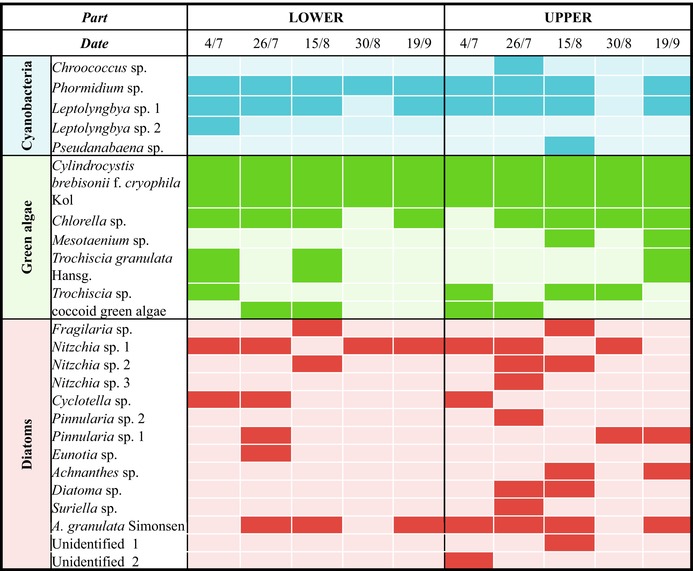

**FIGURE 2:**
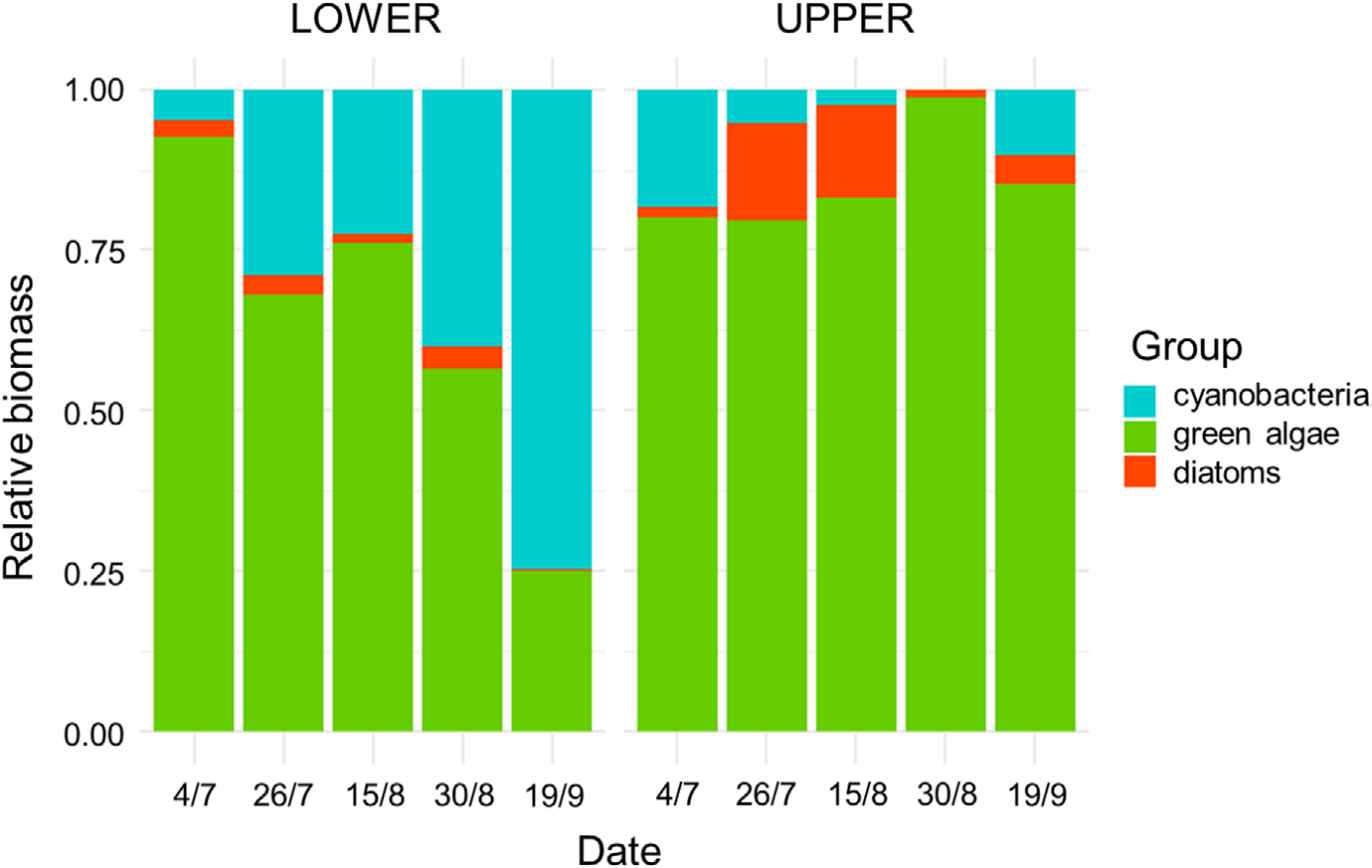
Relative biomass of photoautotrophs (cyanobacteria, green algae and diatoms) in the lower and upper part of the Forni Glacier during the 2019 ablation season.

**FIGURE 3:**
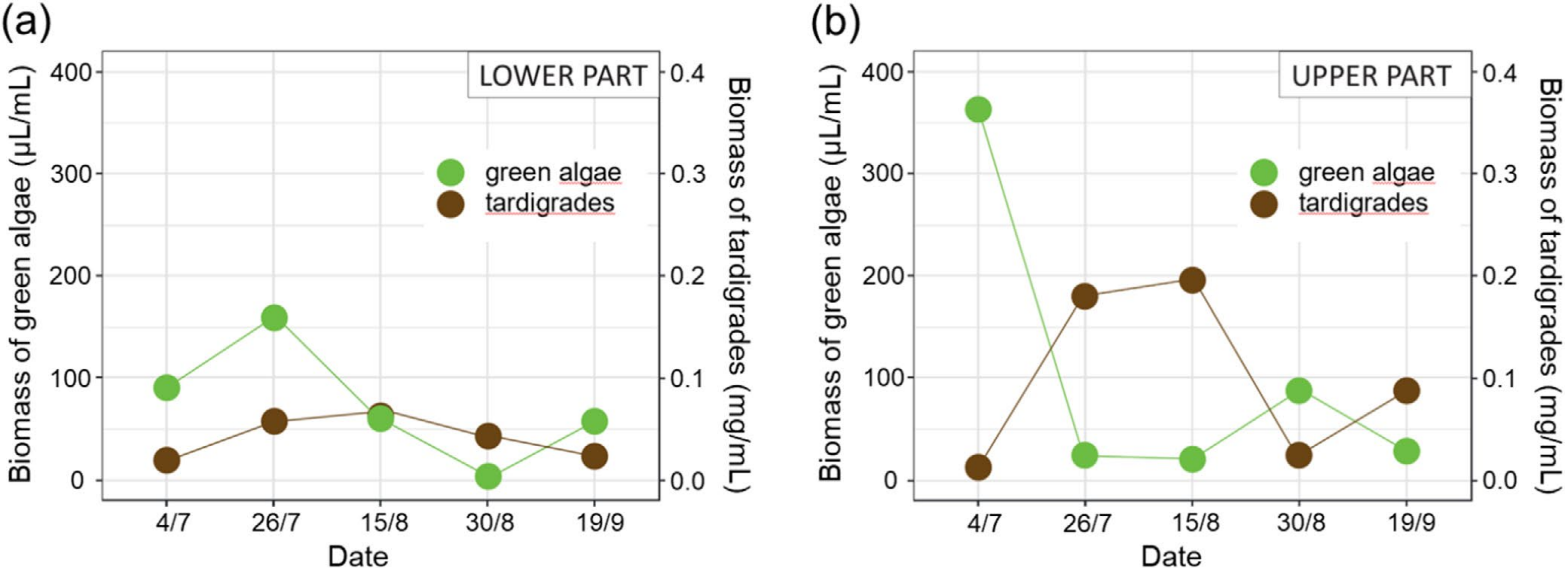
Total biomass of green algae (μL/mL) and tardigrades (mg/mL) in the lower (a) and upper (b) part of the Forni Glacier ablation zone during the 2019 ablation season.

The relative biomass of photoautotrophs varied during the season and differed between the upper and lower parts of the ablation zone (Figure 2). At the beginning of the season (from July 4 to July 26), the total biomass of green algae in the lower part increased (Figure 3a) while that in the upper part decreased (Figure 3b). The total biomass of green algae and all photoautotrophs together was highest on July 4 in the upper part (Table 2; Figure S3).

**TABLE 2:** Biomass of photoautotrophs and tardigrades, the stable isotopic composition of cryoconite and tardigrades, the organic matter content (LOI) in cryoconite and the general elemental composition (< 1000 μg/kg) of cryoconite on the Forni Glacier over the 2019 ablation season.

Part/Date	Group	LOWER					UPPER				
		4/7	26/7	15/8	30/8	19/9	4/7	26/7	15/8	30/8	19/9
Biomass mm^3^/mL mg/mL	*green algae*	89.05	160.10	60.97	3.91	57.40	363.46	24.24	21.14	87.31	28.49
	*cyanobacteria*	4.57	68.21	18.00	2.77	172.17	83.19	1.63	0.64	0	3.43
	*diatoms*	2.5	7.37	1.10	0.24	0.92	7.43	4.58	3.63	1.18	1.46
	*tardigrades*	0.019	0.057	0.063	0.043	0.022	0.014	0.179	0.195	0.024	0.089
LOI (%)	*cryoconite*	5.01	10.15	11.97	10.54	9.34	10.88	10.57	9.67	9.74	10.16
Stable isotopes (‰)	*δ* ^*13*^ *C tardigrades*	−25.62	−25.89	−26.16	x	−23.96	−23.52	−27.15	−27.23	−27.36	−26.98
	*δ* ^*15*^ *N tardigrades*	x	−6.32	−7.14	x	x	x	−7.22	−7.01	x	−7.34
	*δ* ^*13*^ *C cryoconite*	−22.05	−21.4	−21.42	−20.57	−20.18	−21.29	−23.31	−23.37	−23.46	−22.9
		−22.07	−21.41	−21.70	−20.5	−20.12	−21.25	−23.29	−23.37	−23.35	−22.91
		−22.18	−21.41	−21.73	−20.63	−20.18	−21.2	−23.29	−23.31	−22.3	−22.93
		x	x	x	x	−20.29	x	x	x	x	x
	*δ* ^*15*^ *N cryoconite*	−4.86	−5.38	−4.96	−5.13	−4.91	−5.18	−4.68	−4.65	−4.66	−5.52
		−4.78	−5.37	−4.93	−5.16	−4.94	−5.15	−4.73	−4.67	−4.61	−5.53
		−4.86	−5.32	−4.94	−5.21	−4.77	−5.29	−5.21	−4.61	−4.64	x
Elements (mg/kg)	*Ca*	2262.37	3011.65	3131.67	3010.48	2580.36	2212.93	3318.53	3127.72	2926.14	4167.04
	*K*	938.16	1836.39	2489.82	2333.05	2051.71	1824.23	2294.27	1854.77	1975.69	2486.16
	*P*	1475.14	3477.00	5324.68	4375.48	3151.21	4619.07	4435.44	3935.42	1298.59	4289.11
	*Si*	1867.90	4848.49	6121.67	6865.62	5059.43	3970.48	5700.19	4190.48	5159.58	7194.23
	*Al*	3186.38	7904.64	12475.82	11146.5	8580.33	8053.97	11113.9	8990.04	9121.65	8981.54
	*Mg*	978.77	2158.95	3139.39	3106.72	2337.62	2100.21	2880.08	2462.2	2375.31	2311.53

In the lower part of the ablation zone, the relative biomass of cyanobacteria significantly increased during the season (t_6_ = 4.734, *p*_FDR_ = 0.021), the relative biomass of green algae decreased (t_6_ = −4.642, *p*_FDR_ = 0.021) and diatoms were stable (t_6_ = −0.238, *p*_FDR_ = 0.831). In the upper part, no taxon showed any significant trend in the relative biomass (|t_6_| ≤ 1.684, *p* ≥ 0.395).

### 3.2 Consumers in Cryoconite Throughout the Ablation Season

The dominant invertebrates found in cryoconite were tardigrades, represented by a single species, *Cryobiotus klebelsbergi*. Few individuals of bdelloid rotifers were detected, with a very low occurrence (few or no specimens among hundreds of tardigrades); thus, they were not analysed further.

At the beginning of the ablation season, tardigrade biomass was higher in the lower than in the upper part of the ablation zone (Table 2). On average, the total biomass of tardigrades in the upper part did not differ from that of the lower part (t_8_ = 0.854, *p* = 0.418).

### 3.3 The Interaction Between Photoautotrophs and Consumers Throughout the Ablation Season

The biomass of tardigrades in the upper part was significantly and negatively related to the biomass of green algae (Pearson's correlation: r = −0.98; *p* = 0.003). No relation between the biomass of tardigrades and green algae was observed in the lower part of the ablation zone (r = −0.193; *p* = 0.76). Also, no significant relation between the biomass of tardigrades and those of cyanobacteria and diatoms was found in both parts of the ablation zone (sampling areas) (|r| ≤ 0.248, *p* ≥ 0.687).

**FIGURE 4:**
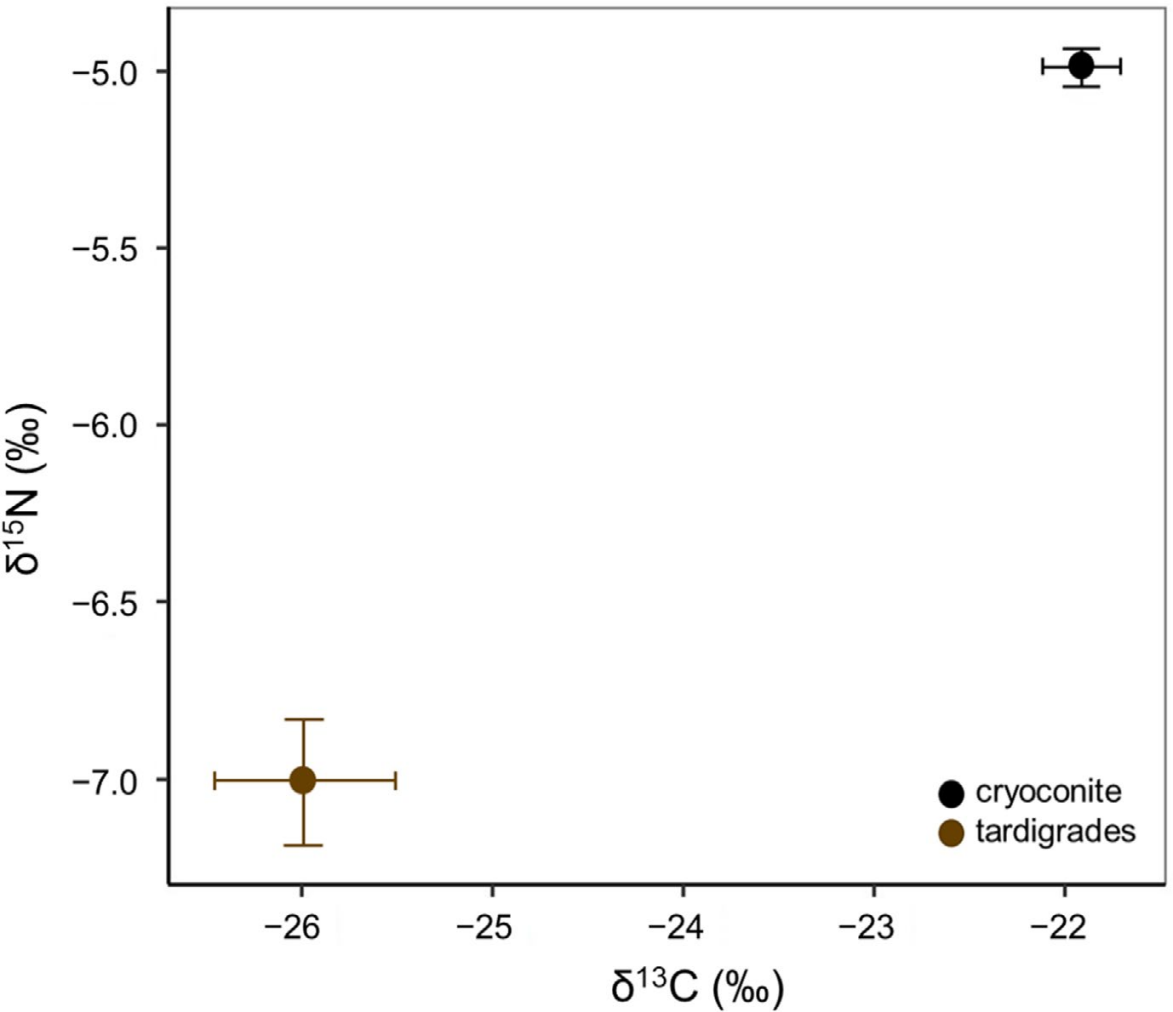
Mean values of δ^13^C and δ^15^N in cryoconite and tardigrades with standard deviation and standard error bars.

Tardigrades were depleted in both δ^13^C and δ^15^N (|t_16_| ≥ 7.157, *p* < 0.001) compared to cryoconite (Figure 4). The δ^13^C of tardigrades significantly and positively correlated with δ^13^C of cryoconite in the upper part (Pearson's correlation: r = 0.93; *p* = 0.02). Further analyses of tardigrade stable isotopes were not feasible due to a low dry weight of some tardigrade samples, making the reliability of their δ^15^N results low.

**FIGURE 5:**
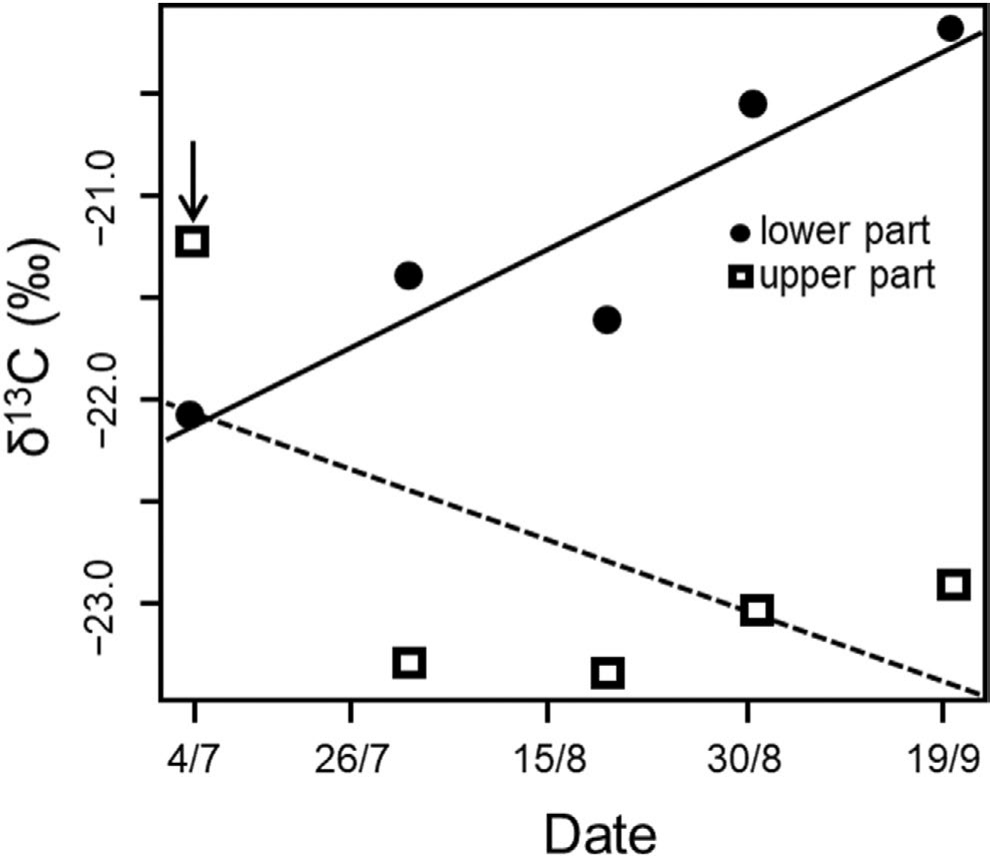
Scatterplot of δ^13^C values in cryoconite in the upper and lower part of the ablation zone. The black arrow indicates an influential point whose removal did not change the results.

**FIGURE 6:**
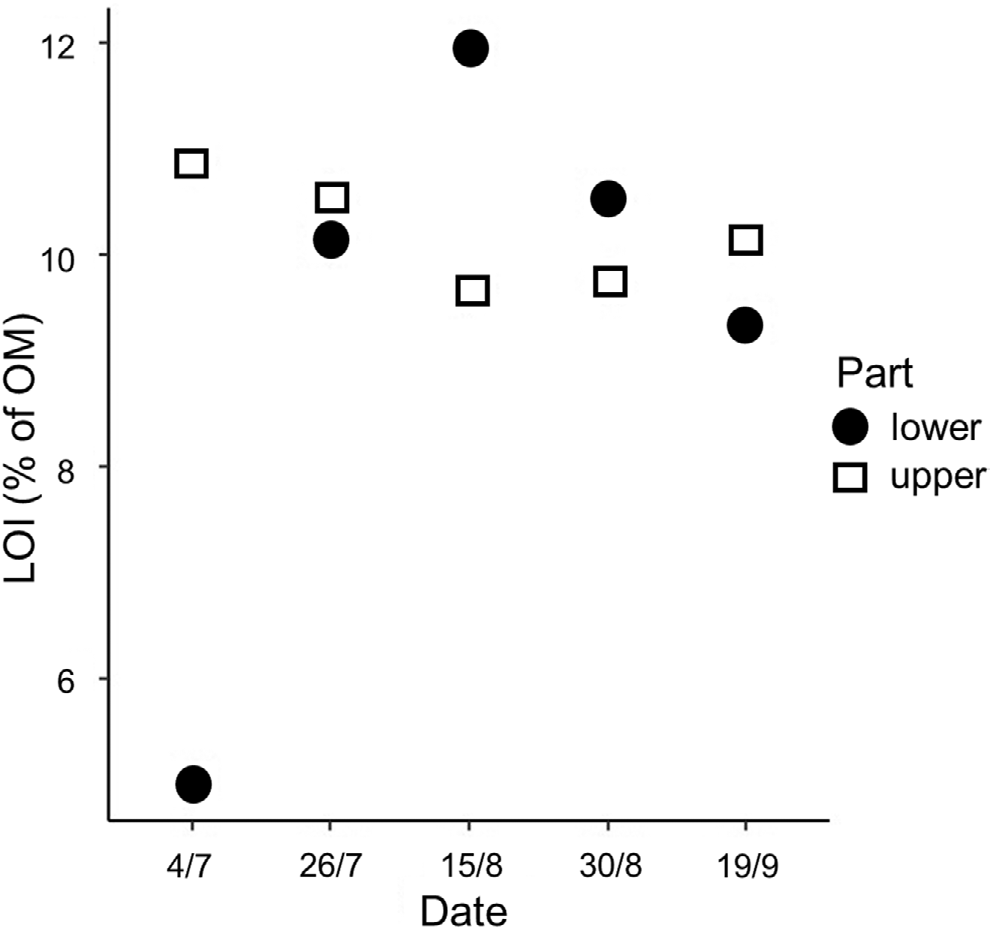
Percentage of organic matter content (LOI) in both parts of the ablation zone over the 2019 ablation season.

A linear model of δ^13^C values revealed that they were, on average, higher in the lower part than in the upper part of the ablation zone (coef. ± SE: 1.612 ± 0.389, t_6_ = 4.144, *p* = 0.006) and changed during the season according to divergent trends in both parts of the ablation zone (sampling area by day‐of‐year interaction, F_1,6_ = 8.237, *p* = 0.029; Figure 5). In particular, the δ^13^C values increased (i.e., samples were enriched in ^13^C) during the ablation season in the lower part of the ablation zone (coef. ± SE: 0.024 ± 0.010, t_6_ = 2.363, *p* = 0.045) while no relationship was observed for the upper part (coef. ± SE: −0.017 ± 0.010, t_6_ = −1.696, *p* = 0.141, Figure 5), even when the first and most enriched value was removed (t_5_ = 1.196, *p* = 0.285). No significant variation of δ^15^N of OM in cryoconite was observed (F_3,6_ = 3.150, *p* = 0.108). The OM content did not vary between the upper or the lower part of the ablation zone over the ablation season (F_3,6_ = 1.238, *p* = 0.375; Figure 6).

An RDA model on standardised elemental abundances of general elements (Ca, K, P, Si, Al, Mg) showed that their composition did not vary significantly according to date, sampling area, or their interaction (overall model: *F*_3,6_ = 0.305, *p* = 0.937). The summary of all data is provided in Table 2. The abundance and concentration of all analysed elements are shown in Figure S4.

## 4 Discussion

### 4.1 Dynamics of Cryoconite Biota From the Lower Part of the Ablation Zone Throughout the Ablation Season

Meltwater may be an important factor in the redistribution of cryoconite and supraglacial organisms, along with nutrients, on glacier surfaces (Dubnick et al. 2017; Takeuchi 2001; Takeuchi et al. 2019; Zawierucha, Buda, and Nawrot 2019) and repeated pulses of the meltwater at the beginning of the ablation season might have increased the OM content together with the biomass of photoautotrophs (mostly green algae) and tardigrades in the lower part of the Forni Glacier ablation zone.

During the season, we observed a shift in the community structure of photoautotrophs from a dominance of green algae (Zygnemataceae and Chlorellaceae) to a dominance of cyanobacteria (Oscillatoriaceae and Leptolyngbyaceae). Cyanobacteria are known for their adaptations (e.g., N‐fixation) allowing them to thrive on glacier surfaces and colonise nutrient‐poor areas such as glacier debris or areas where the input of nutrients from adjacent areas is low (Stibal et al. 2006; Telling et al. 2012). The shift from algae to cyanobacteria has also been observed in Antarctic cyanobacterial mats along with the increasing temperature, removal of ice coverage and the shift in the nutrient (mostly N and P) pool (Velázquez et al. 2017). Cryoconite holes on the Forni Glacier are predominantly without ice lids compared to those in Antarctica, and their temperature oscillates at around freezing during the whole ablation season. We also did not observe any considerable increase in δ^15^N which might indicate higher N‐fixation and low nitrogen availability on the glacier surface (e.g., Schmidt et al. 2022). Thus, we assume that the cyanobacterial growth in the lower part was mainly supported by the high supraglacial debris coverage (Uetake et al. 2016; Zawierucha et al. 2019b; Wejnerowski et al. 2023). Also, the firm attachment of cyanobacterial communities to the substrate might help them withstand the flush‐out from the glacier surface during precipitation (Dubnick et al. 2017). The meteorological data corroborate higher precipitation in August (Figure S1) which may have contributed to the flush‐out of green algae and also supported holes with dissolved inorganic nitrogen (DIN) (Dubnick et al. 2017).

Since δ^13^C of cryoconite OM seems to be triggered by its dominant compound (Novotná Jaroměřská et al. 2021, 2023), the shift in the photoautotrophic community structure likely contributes to the increasing trend in δ^13^C of cryoconite OM. Cyanobacteria are significant primary producers in cryoconite (e.g., Säwström et al. 2002; Stibal et al. 2012b) which could result in the enrichment of δ^13^C from the uptake of isotopically heavier (depleted in ^12^C) dissolved inorganic carbon (DIC) which is preferred by cryoconite producers (Musilova et al. 2015; Stibal and Tranter 2007).

Compared to cryoconite with photosynthetically active cyanobacteria in Continental Antarctica (Schmidt et al. 2022), OM from the Forni Glacier has low δ^13^C indicating high input of allochthonous matter (Azzoni et al. 2016). Even though we expect that allochthonous OM and external forces such as meltwater (triggered by meteorological conditions) affect cryoconite in the lower part of the ablation zone to a greater extent compared with the upper part, the density of photosynthetically active cells can remain high and considerably influence the overall δ^13^C values (Musilova et al. 2015).

Consumers remained almost stable with a slight increase in their biomass at the beginning of the season and a slight decrease at the end. Higher melt‐water dynamics increasing instability of cryoconite in holes (interhole water‐sediment mixing) in the lower part likely did not allow consumers to increase their biomass as fast as observed in the upper part (Zawierucha et al. 2019a). However, more samples collected during the ablation season could help to explain the observed processes. The consumption of cyanobacteria by tardigrades is a taxon‐dependent feature which is avoided while preferable sources (i.e., algae) are available (Bryndová et al. 2020; Zawierucha et al. 2022). Therefore, both physical conditions and available food sources likely affect the biomass of tardigrades in the lower part.

### 4.2 Dynamics of Cryoconite Biota From the Upper Part of the Ablation Zone Throughout the Ablation Season

In the upper part of the Forni Glacier ablation zone, green algae (mostly Zygnemataceae and Chlorellaceae) dominated the community structure of photoautotrophs and their total biomass negatively correlated with the biomass of tardigrades along the season. Algae are known for their fast growth (Lürling et al. 2013; Nalley et al. 2018), which allows them to dominate communities of photoautotrophs in cryoconite holes (Uetake et al. 2010; Stibal et al. 2012b; Buda et al. 2020) if other factors (e.g., meltwater pulse) do not disturb their occurrence. Also, the concentration of elements such as Ca (essential for tardigrades during moulting) or Si (essential for diatoms) could affect the community structure of supraglacial biota (e.g., Uetake et al. 2010; Guidetti et al. 2015). Our results indicate that biological control likely drives the structure of cryoconite biota over the season in the upper part, with the positive effect of grazing on the growth of green algae. This conclusion is partially supported by the absence of significant differences in the general elemental composition in cryoconite between the two parts of the ablation zone. However, it should be noted that the same composition can be a result of the local origin of elements such as Si, Al, Ca, Mg and P from nearby mountains, and the effect of limited sample size has to be considered.

The field and laboratory observations of *C. klebelsbergi* confirmed that this species actively feeds on green algae (Zawierucha et al. 2022, 2023). The same observation of feeding behaviour was reported for tardigrades with similar buccal tube morphology in other studies (e.g., Bryndová et al. 2020; Roszkowska et al. 2021). Also, at the beginning of the season, a rapid increase in the biomass of tardigrades was followed by a rapid decrease in the total biomass of algae. The only date when we observed a reduction in the biomass of tardigrades accompanied by an increase in green algae total biomass coincided with the presence of numerous small tardigrade juveniles (K. Zawierucha pers. observ.) indicating lower grazing pressure favouring the algal growth. Owing to the complexity of cryoconite granules, we were not able to analyse each compartment of cryoconite for isotopic composition separately to focus on the exact food of consumers. However, δ^13^C values from the upper part corroborate the previous assumption on the high influence of bulk cryoconite OM δ^13^C by the dominant OM compound. Also, the influential point which marks the highest total biomass of algae (and photoautotrophs) is for the same date when the highest δ^13^C of cryoconite OM was measured. In the upper part, δ^13^C of cryoconite OM significantly correlated with δ^13^C of tardigrades. Such a trend supports the grazing control over the total biomass of green algae since no correlation between consumers and cryoconite OM was observed in the lower part where cyanobacteria dominated the photoautotroph community structure.

## 5 Conclusions

In this study, we described patterns in the biomass, community structure and stable carbon and nitrogen isotopic composition of biota from cryoconite holes in the lower and upper parts of the Forni Glacier ablation zone during the 2019 ablation season. Changes in the community structure and biomass of photoautotrophs and consumers (tardigrades) in both parts of the ablation zone indicated phenological or ecological controls over their communities which can be further affected by environmental conditions. Based on our data, photoautotrophs in cryoconite holes in the upper part of the ablation zone are likely influenced by their consumers. However, the higher dynamics (e.g., effects of meltwater disturbances) in cryoconite holes may lower the grazing pressure which results in higher variability in the community structure of photoautotrophs as observed in the lower part of the ablation zone where the photoautotrophs shifted from algae‐ to cyanobacteria‐dominated during the season. Moreover, some photoautotrophs appeared only during specific dates, highlighting that rare species might be overlooked during single sampling campaigns.

We cannot exclude influence of other factors such as meltwater, weathering and the input of matter from adjacent sources that require further investigation in studies on the development of communities in cryoconite holes throughout the ablation season. However, we have demonstrated that understanding the ecology of biota in cryoconite holes requires a broad‐scale and seasonal approach.

## Author Contributions

**Tereza Novotná Jaroměřská:** data curation (equal), formal analysis (equal), funding acquisition (equal), investigation (equal), methodology (equal), resources (equal), writing – original draft (lead), writing – review and editing (equal). **Roberto Ambrosini:** data curation (equal), formal analysis (lead), investigation (equal), methodology (equal), writing – original draft (equal), writing – review and editing (equal). **Dorota Richter:** investigation (equal), resources (equal), writing – review and editing (equal). **Miroslawa Pietryka:** investigation (equal), resources (equal), writing – review and editing (equal). **Przemyslaw Niedzielski:** investigation (equal), resources (equal), writing – review and editing (equal). **Juliana Souza‐Kasprzyk:** investigation (equal), resources (equal), writing – review and editing (equal). **Piotr Klimaszyk:** investigation (equal), resources (equal), writing – review and editing (equal). **Andrea Franzetti:** investigation (equal), writing – review and editing (equal). **Francesca Pittino:** investigation (equal), writing – review and editing (equal). **Lenka Vondrovicová:** formal analysis (equal). **Antonella Senese:** investigation (equal), resources (equal), writing – review and editing (equal). **Krzysztof Zawierucha:** conceptualization (equal), data curation (equal), funding acquisition (equal), investigation (equal), methodology (equal), project administration (lead), resources (equal), supervision (lead), writing – original draft (lead), writing – review and editing (lead).

## Conflicts of Interest

The authors declare no conflicts of interest.

## Supporting information


Figure S1.

Figure S2.

Figure S3.

Figure S4.

Data S1.

Table S1.

Data S2.

## Data Availability

Data used in the manuscript are presented in supplementary files. Raw data are available upon request to TNJ and KZ.
