# Supplementary material for: Insights Into Cryoconite Community Dynamics on the Alpine Glacier Throughout the Ablation Season
Source: Ecol Evol. 2025 Mar 24;15(3):e71064. doi: 10.1002/ece3.71064 (PMC11932729; doi:10.1002/ece3.71064)

Insights into cryoconite community dynamics on the alpine glacier throughout the ablation season

Tereza Novotná Jaroměřská, Roberto Ambrosini, Dorota Richter, Miroslawa Pietryka, Przemyslaw Niedzielski, Juliana Souza-Kasprzyk, Piotr Klimaszyk, Andrea Franzetti, Francesca Pittino, Lenka Vondrovicová, Antonella Senese, Krzysztof Zawierucha

Figure S1. Meteorological conditions at the Forni Glacier from June 1st to September 30th 2019. A) Modelled and measured daily air temperature (the modelled series is derived from Santa Caterina Valfurva data shifted to the AWS1 Forni elevation through the application of daily lapse rate). B) Daily wet precipitation acquired at Santa Caterina Valfurva and AWS1 Forni site.

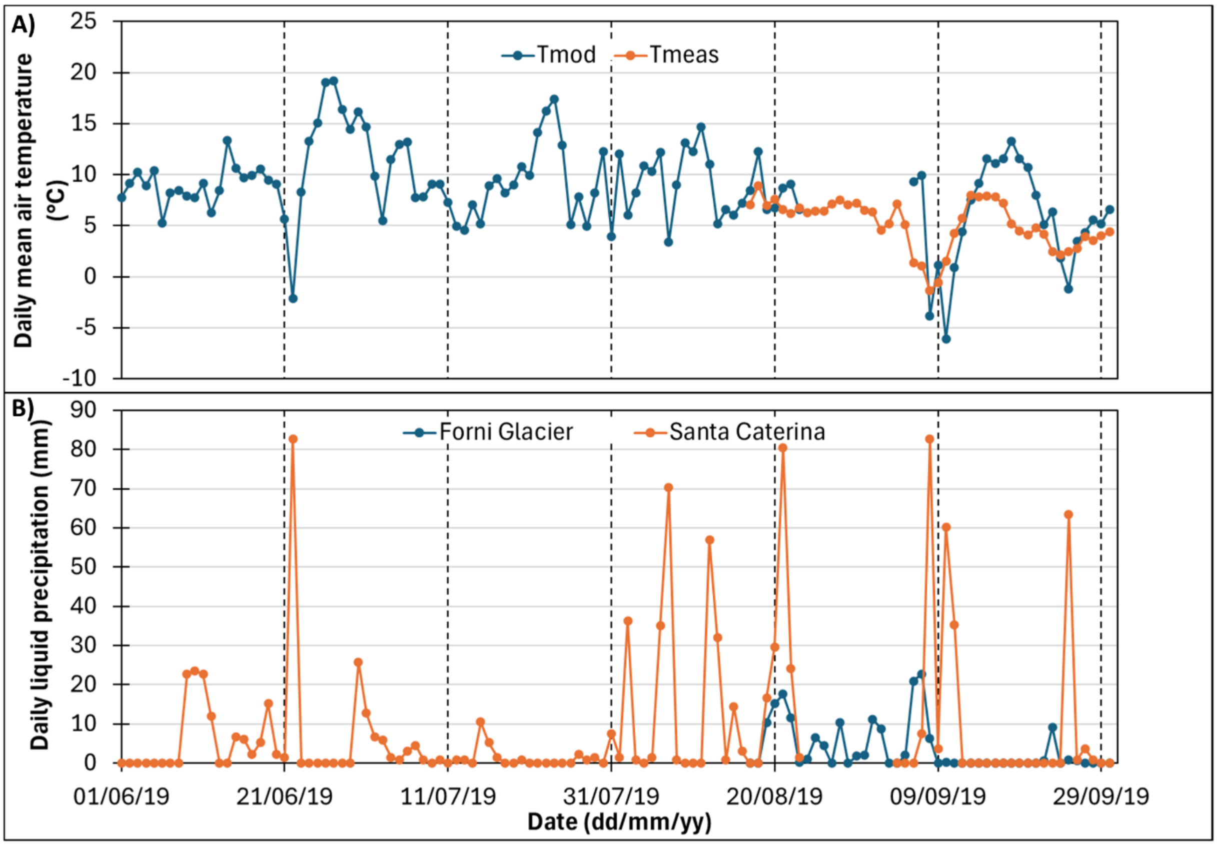

Supplement: Supplementary file 1 — Figure S1. [file ECE3-15-e71064-s001.pdf]
