# Supplementary material for: Insights Into Cryoconite Community Dynamics on the Alpine Glacier Throughout the Ablation Season
Source: Ecol Evol. 2025 Mar 24;15(3):e71064. doi: 10.1002/ece3.71064 (PMC11932729; doi:10.1002/ece3.71064)

Insights into cryoconite community dynamics on the alpine glacier throughout the ablation season

Tereza Novotná Jaroměřská, Roberto Ambrosini, Dorota Richter, Miroslawa Pietryka, Przemyslaw Niedzielski, Juliana Souza-Kasprzyk, Piotr Klimaszyk, Andrea Franzetti, Francesca Pittino, Lenka Vondrovicová, Antonella Senese, Krzysztof Zawierucha

Figure S2. Cyanobacteria, green algae, and diatoms found on the Forni Glacier. 1. *Mesotaenium* sp., 2. *Chlorella* sp., 3. *Cylindrocystis brebisonii* f. *cryophila* Kol, 4. *Trochiscia granulata* Hansg., 5. *Trochiscia* sp., 6. *Phormidium* sp., 7. *Leptolyngbya* sp.

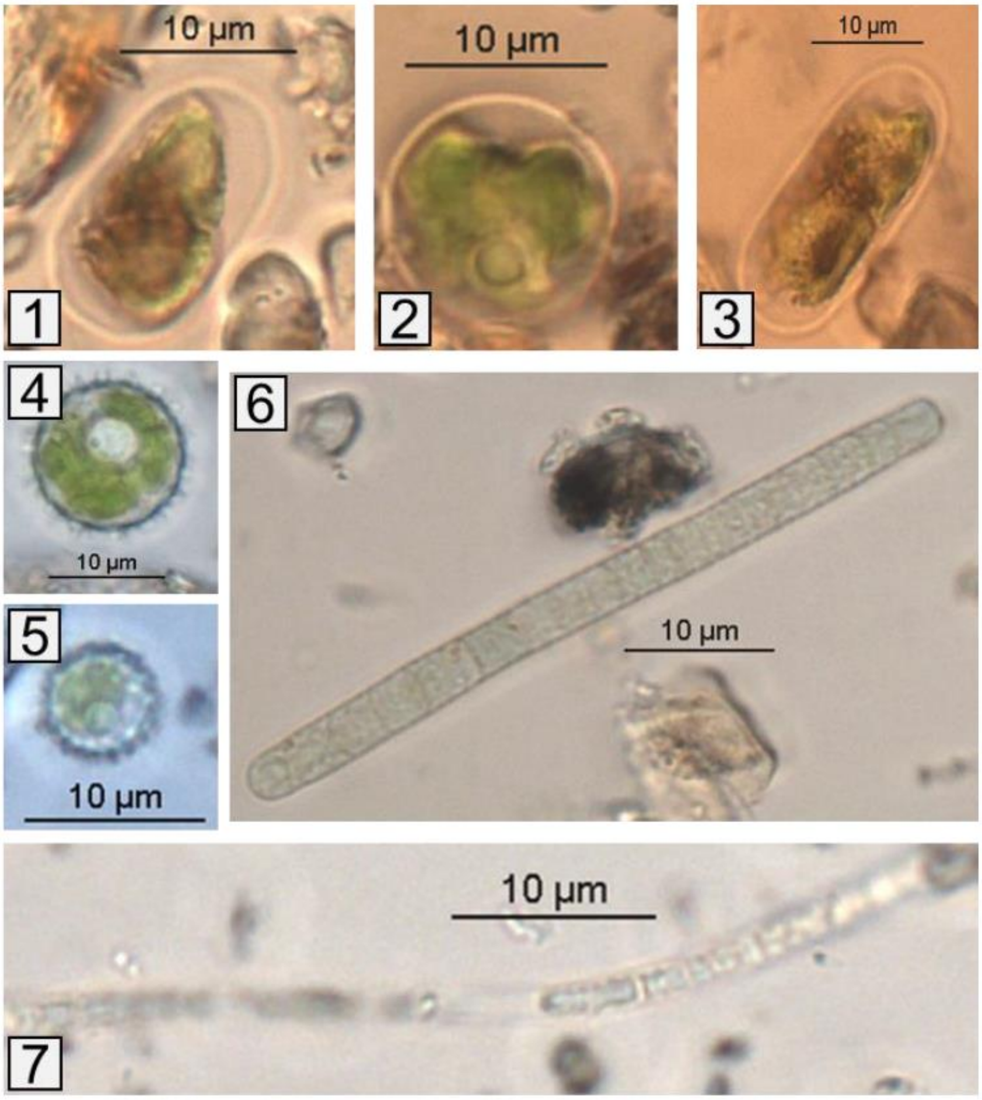

Supplement: Supplementary file 2 — Figure S2. [file ECE3-15-e71064-s007.pdf]
