# Supplementary material for: Insights Into Cryoconite Community Dynamics on the Alpine Glacier Throughout the Ablation Season
Source: Ecol Evol. 2025 Mar 24;15(3):e71064. doi: 10.1002/ece3.71064 (PMC11932729; doi:10.1002/ece3.71064)

Insights into cryoconite community dynamics on the alpine glacier throughout the ablation season

Tereza Novotná Jaroměřská, Roberto Ambrosini, Dorota Richter, Miroslawa Pietryka, Przemyslaw Niedzielski, Juliana Souza-Kasprzyk, Piotr Klimaszyk, Andrea Franzetti, Francesca Pittino, Lenka Vondrovicová, Antonella Senese, Krzysztof Zawierucha

Figure S3. Total biomass of photoautotrophs (cyanobacteria, green algae, and diatoms) in the lower and upper part of the Forni Glacier during the 2019 ablation season.

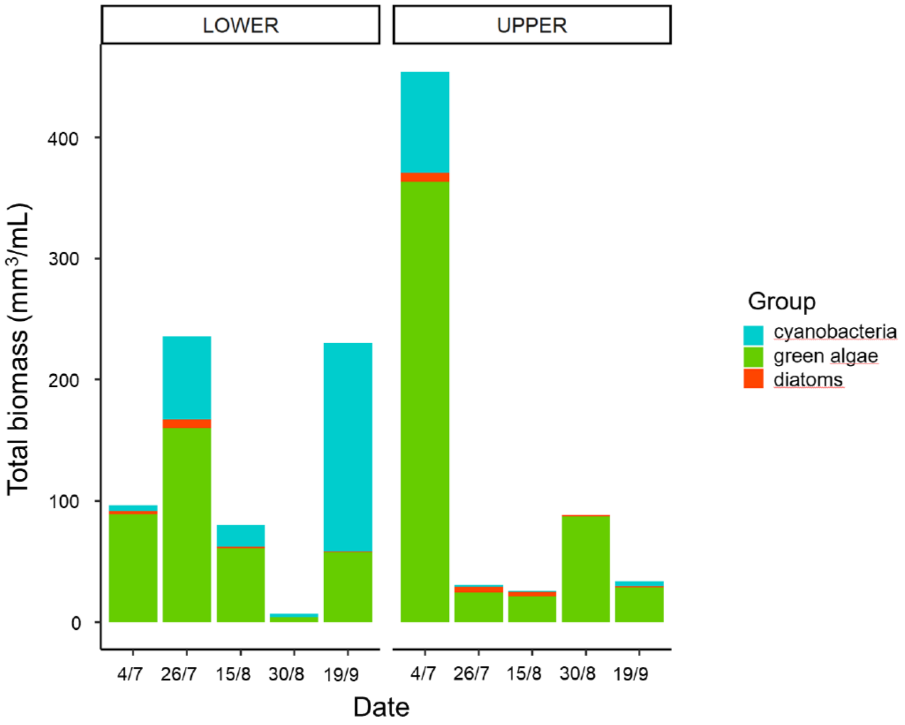

Supplement: Supplementary file 3 — Figure S3. [file ECE3-15-e71064-s005.pdf]
