# Supplementary material for: Insights Into Cryoconite Community Dynamics on the Alpine Glacier Throughout the Ablation Season
Source: Ecol Evol. 2025 Mar 24;15(3):e71064. doi: 10.1002/ece3.71064 (PMC11932729; doi:10.1002/ece3.71064)

Insights into cryoconite community dynamics on the alpine glacier throughout the ablation season

Tereza Novotná Jaroměřská, Roberto Ambrosini, Dorota Richter, Miroslawa Pietryka, Przemyslaw Niedzielski, Juliana Souza-Kasprzyk, Piotr Klimaszyk, Andrea Franzetti, Francesca Pittino, Lenka Vondrovicová, Antonella Senese, Krzysztof Zawierucha

Figure S4. Elemental composition of cryoconite in the lower and the upper part of ablation zone on Forni Glacier in 2019.

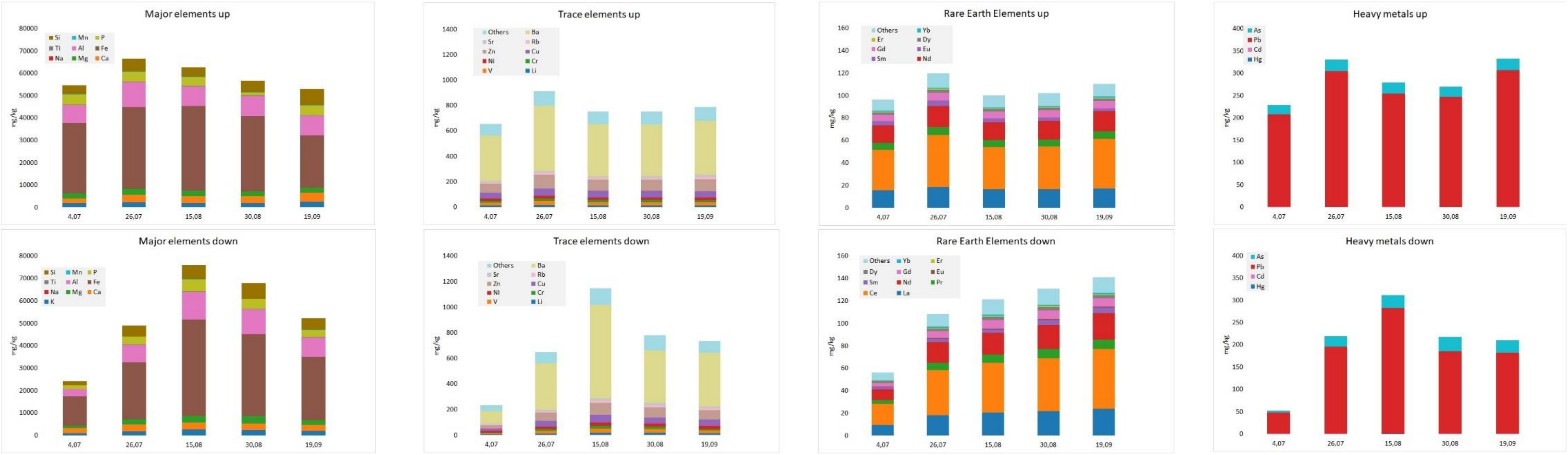

Supplement: Supplementary file 4 — Figure S4. [file ECE3-15-e71064-s006.pdf]
