# Supplementary material for: Insights Into Cryoconite Community Dynamics on the Alpine Glacier Throughout the Ablation Season
Source: Ecol Evol. 2025 Mar 24;15(3):e71064. doi: 10.1002/ece3.71064 (PMC11932729; doi:10.1002/ece3.71064)
Supplement: Supplementary file 5 — Data S1. [file ECE3-15-e71064-s002.pdf]

Supplementary methods

Insights into cryoconite community dynamics on the alpine glacier throughout the ablation season

Tereza Novotná Jaroměřská, Roberto Ambrosini, Dorota Richter, Miroslawa Pietryka, Przemyslaw Niedzielski, Juliana Souza-Kasprzyk, Piotr Klimaszyk, Andrea Franzetti, Francesca Pittino, Lenka Vondrovicová, Antonella Senese, Krzysztof Zawierucha

Meteorological conditions at Forni Glacier from June 1st to September 30th 2019

For characterizing meteorological conditions during the ablation season, we considered sub-hourly data of air temperature (Tsc) and liquid precipitation (Psc) acquired by a private weather station located at Santa Caterina Valfurva (1730 m a.s.l.) from June 1st to September 30th 2019 (with a unique gap from August 24th to September 5th). We could not use the data acquired directly on the glacier by the supraglacial automatic weather station AWS1 Forni (2675 m a. s. l.) due to technical problems which caused that data were not acquiredin the first part of the ablation season (until August 16th 2019). Unfortunately, also the meteorological station near the Forni Glacier managed by the Lombardy Regional Agency for Environmental Protection (ARPA) and located at Santa Caterina Valfurva did not work in summer 2019.

Following Senese et al. (2016) to estimate the thermal regime at the Forni Glacier (Tmod), we applied a daily lapse rate to daily mean Tsc. To avoid thermal inversion to affect the data too much, only the warmest hours of the day (from 11AM to 3PM) were considered in the calculation of the daily averages. Indeed, in a previous study (Senese et al., 2014) Santa Caterina Valfurva was found to be affected by thermal inversion for about 9% of the period from 2006 to 2012. We checked the feasibility of applying this approach by comparing the daily air temperatures measured by the AWS1 Forni (Tmeas) with the modelled ones. We found a bias (BE) of −0.6°C, a mean absolute error (MAE) of 2.7°C, a root mean square error (RMSE) of 3.6°C, and a bias-removed root mean square error (BRRMSQ) of 3.5°C. Although these errors are slightly higher than the results of the previous study (Senese et al., 2016), both series seem to have the same pattern (Fig. S1). Considering the modelled values, we found a mean daily air temperature of +8.7°C, ranging from −6.1°C to +19.2°C.

Regarding wet precipitation, data acquired by the two weather stations (at Santa Caterina and Forni Glacier) are almost in agreement (Fig. S1): for 76% of days both stations recorded a rain or a no rain event (totally 34 days). By taking into the account albedo (i.e. the bihemispherical reflectance, calculated as the ratio of the reflected solar radiation to the incident solar radiation), we can detect the snowfall (Senese et al., 2021). From June 1st to September 30th 2019, considering both precipitation and albedo series, there were 61 rainy days (50% of the period). Certainly, since August 16th there were 2 snowfalls resulting in the glacier surface covered by snow for a total of 10 days. Both snowfall events occurred with daily air temperatures of about 1–2°C (Fig. S1). This agrees with the temperature threshold value of +1.5°C to distinguish snowfall from wet precipitation (Senese et al., 2012). Considering also this temperature criterion, the rainfall of June 22nd detected at Santa Caterina could be a snowfall at the Forni Glacier. Unfortunately, we cannot derive the albedo values of the first part of the period from the Santa Caterina station, as the albedo depends on the characteristics of the investigated surface (Azzoni et al., 2016). Therefore, we are not able to correctly distinguish snowfall from rain events from June 1st to August 16th 2019.

References

Senese, A., Diolaiuti, G., Mihalcea, C., Smiraglia, C., 2012. Energy and mass balance of forni glacier (Stelvio National Park, Italian Alps) from a four-year meteorological data record. Arct Antarct Alp Res 44. https://doi.org/10.1657/1938-4246-44.1.122

Senese, A., Leidi, M., Diolaiuti, G., 2021. A NEW ENHANCED TEMPERATURE-INDEX MELT MODEL INCLUDING NET SOLAR AND INFRARED RADIATION. Geografia Fisica e Dinamica Quaternaria 44. https://doi.org/10.4461/GFDQ.2021.44.1

Senese, A., Maugeri, M., Ferrari, S., Confortola, G., Soncini, A., Bocchiola, D., Diolaiuti, G., 2016. Modelling shortwave and longwave downward radiation and air temperature driving ablation at the Forni Glacier (Stelvio National Park, Italy). Geografia Fisica e Dinamica Quaternaria 39. https://doi.org/10.4461/GFDQ.2016.39.9

Senese, A., Maugeri, M., Vuillermoz, E., Smiraglia, C., Diolaiuti, G., 2014. Using daily air temperature thresholds to evaluate snow melting occurrence and amount on Alpine glaciers by T-index models: The case study of the Forni Glacier (Italy). Cryosphere 8. https://doi.org/10.5194/tc-8-1921-2014

Azzoni, R.S., Senese, A., Zerboni, A., Maugeri, M., Smiraglia, C., Adele Diolaiuti, G., 2016. Estimating ice albedo from fine debris cover quantified by a semi-automatic method: The case study of Forni Glacier, Italian Alps. Cryosphere 10. https://doi.org/10.5194/tc-10-665-2016

Algae identification

Identification was conducted using NIS image analysis software. Identification of cyanobacteria, green algae and diatoms (only specimens with well-preserved, visible chloroplasts were considered for analyses) followed Krammer and Lange-Bertalot (1986, 1991a, 1991b), Hindak (1996), Komárek and Anagnostidis (2005), Coesel and Meesters (2007), John and Rindi (2015).

References

Coesel, P. F. and Meesters, K. J. 2007. Mesotaeniaceae and Desmidiaceae of the European Lowlands. Desmids of the Lowlands, KNNV Publishing, Leiden, Netherlands, 351 pp.

Hindak, F. 1996. Key to the unbranched filamentous green algae (Ulotrichineae, Ulotrichales, Chlorophyceae). Bulletin Slovenskej botanickej spoločnosti pri SAV, Bratislava, Slovakia, 77 pp.

John, D. M. and Rindi, F. 2015. Filamentous (Nonconjugating) and Plantlike Green Algae. Wehr, J. D., Sheath, R. G., Kociolek, J. P. (Eds.): Freshwater algae of North America: Ecology and classification, Academic, San Diego, U. S., 375–427 pp.

Komárek, J. and Anagnostidis, K. 2005. Cyanoprokaryota. 2. Teil: Oscillatoriales. Büdel, B., Gärtner, G., Krienitz, L., Schagerl, M. (Eds.): Süßwasserflora von Mitteleuropa, Bd. 19 (2), Elsevier GmbH, München, Germany, 1−759 pp.

Krammer, K. and Lange-Bertalot, H. 1986. Bacillariophyceae 1. Naviculaceae. Suβwassweflora von Mitteleuropa, Gustav Fisher Verlag, Stuttgart, Germany, 1−876 pp.

Krammer, K. and Lange-Bertalot, H. 1991a. Bacillariophyceae 2/3. Centrales, Fragilariaceae, Eunotiaceae. Ettl, H., Gerloff, J., Heynig, H., Mollenhauer, D. (Eds.). Süβwasserflora von Mitteleuropa, Gustav Fischer, Stuttgart, Germany, 1−576 pp.

Krammer, K. and Lange-Bertalot, H. 1991b. Bacillariophyceae 4. Achnanthaceae, Kritische Ergänzungen zu *Navicula* (Lineolatae), *Gomphonema* Gesamtliteraturverzeichnis Teil 1–4. Ettl, H., Gärtner, G., Gerloff, J., Heynig, H., Mollenhauer, D. (Eds.), Suesswasserflora von Mitteleuropa 2, Spektrum Akademischer Verlag, Heidelberg, Germany, 1–468 pp.

Elemental composition

Samples were dried at +35 ± 2 ºC in an electric oven (Thermocenter, Salvislab, Switzerland). Then, 200 mg (with accuracy ± 1 mg) of each sample were extracted in a closed Teflon container with 5 mL of 65% nitric acid (Sigma-Aldrich, USA) using a Mars 6 (Mars 6 Xpress, CEM USA) microwave digestion system. Thereafter, each sample was filtered and refilled to a total volume of 15 mL with Milli-Q water (Direct-Q system, Millipore, Germany). Just before the analysis, each sample was diluted 20 times with Milli-Q water.

The instrumental conditions were: plasma gas flow 9.0 L min^{-1}, auxiliary gas flow 1.5 L min^{-1}, nebulizer gas flow 1.05 L min^{-1}, Radio Frequency (RF) power 1.35 kW, the signal was measured in 5 replicates (20 scans each). The mass interferences were reduced using the integrated Collision Reaction Cell (iCRC) working sequentially in three modes: without gas addition, with hydrogen as reaction gas and with helium as collision gas.

The uncertainty for the total analytical procedure was below 20%. An expanded uncertainty with a coverage factor of k = 2 (approximate 95% confidence) was calculated for all analytical steps including sample preparation and instrumental analysis. The detection limits were calculated as the concentration corresponding to the signal equal to three times the standard deviation of the blank signal at the level of 0.001 mg kg^{-1} of dry weight (DW). The traceability was checked by the analysis of Standard Reference Materials (SRMs) NCS DC73349 (bush branches and leaves, NCS Testing Technology, China), IAEA-405 (estuarine sediments, International Atomic Energy Agency, IAEA, Austria), SRM 2709a (San Joaquin soil, National Institute of Standards and Technology, USA); BCR-667 (estuarine sediments, Institute for Reference Materials and Measurements, Belgium). Adhering to the quality control requirements, the validity of the analysis was determined based on the recovery results of CRMs (certified reference material), which were expected to fall in the range of 80% to 120%. For elements without certified standards, the standard addition method was employed.
