# Supplementary material for: Insights Into Cryoconite Community Dynamics on the Alpine Glacier Throughout the Ablation Season
Source: Ecol Evol. 2025 Mar 24;15(3):e71064. doi: 10.1002/ece3.71064 (PMC11932729; doi:10.1002/ece3.71064)
Supplement: Supplementary file 6 — Table S1. [file ECE3-15-e71064-s004.pdf]

Insights into cryoconite community dynamics on the alpine glacier throughout the ablation season

Tereza Novotná Jaroměřská, Roberto Ambrosini, Dorota Richter, Miroslawa Pietryka, Przemyslaw Niedzielski, Juliana Souza-Kasprzyk, Piotr Klimaszyk, Andrea Franzetti, Francesca Pittino, Lenka Vondrovicová, Antonella Senese, Krzysztof Zawierucha

Table S1. Species composition and biomass of specific algae taxa in lower and upper part of ablation zone on Forni Glacier in 2019.

**Species mm3/L**	**Date of sampling**									
	4.7Down	4.7Up	26.7Down	26.7Up	15.8Down	15.8Up	30.8Down	30.8Up	19.9Down	19.9Up
**Cyanobacteria**										
cf. *Chroococcus* sp.	0	0	0	26,5	0	0	0	0	0	0
Phormidium sp.	3331,3	81173,0	67982,6	1144,1	17441,5	560,8	2770,4	0,0	171868,8	2658,3
Leptolyngbya sp. 1	1047,8	2015,0	224,3	455,6	560,7	38,5	0,0	0,0	304,9	772,7
Leptolyngbya sp. 2	190,1	0,0	0,0	0,0	0,0	0,0	0,0	0,0	0,0	0,0
Pseudanabaena sp.	0,0	0,0	0,0	0,0	0,0	44,2	0,0	0,0	0,0	0,0
Total	**4569,2**	**83188,0**	**68206,8**	**1626,2**	**18002,2**	**643,5**	**2770,4**	**0,0**	**172173,7**	**3431,0**
**Chlorophyta**										
Cylindrocystis brebisoni (Ralfs) De Bary f. cryophila Kol	43535,8	361396,3	148021,8	4011,2	37470,2	4343,8	3913,3	75918,7	54884,5	4725,3
Chlorella sp.	42544,5	0,0	11613,2	17978,1	20970,8	15633,2	0,0	10049,9	2512,5	17754,8
Mesotaenium sp.	0,0	0,0	0,0	0,0	0,0	491,3	0,0	0,0	0,0	737,0
Trochiscia granulata (Reinsch) Hansg.	2638,7	0,0	0,0	0,0	1754,7	0,0	0,0	0,0	0,0	5277,3
Trochiscia sp.	334,3	668,5	0,0	0,0	0,0	668,5	0,0	1337,1	0,0	0,0
coccoid green algae	0,0	1391,6	463,9	2254,5	774,7	0,0	0,0	0,0	0,0	0,0
Total	**89053,3**	**363456,5**	**160098,9**	**24243,8**	**60970,3**	**21136,8**	**3913,3**	**87305,6**	**57397,0**	**28494,5**
**Bacillariophyceae**										
Fragilaria sp.	0,0	0,0	0,0	0,0	177,0	177,0	0,0	0,0	0,0	0,0
Nitzchia sp. 1	300,0	900,0	540,0	1050,0	0,0	0,0	240,1	300,0	300,0	0,0
Nitzchia sp. 2	0,0	0,0	0,0	400,0	300,0	600,0	0,0	0,0	0,0	0,0
Nitzchia sp. 3	0,0	0,0	0,0	100,0	0,0	0,0	0,0	0,0	0,0	0,0
Cyclotella sp.	2200,0	5500,0	4400,0	0,0	0,0	0,0	0,0	0,0	0,0	0,0
Pinnularia sp. 2	0,0	0,0	0,0	800,0	0,0	0,0	0,0	0,0	0,0	0,0
Pinnularia sp. 1	0,0	0,0	440,0	0,0	0,0	0,0	0,0	880,0	0,0	440,0
Eunotia sp.	0,0	0,0	1059,2	0,0	0,0	0,0	0,0	0,0	0,0	0,0
cf. *Achnanthes* sp.	0,0	0,0	0,0	0,0	0,0	1000,0	0,0	0,0	0,0	500,0
Diatoma sp.	0,0	0,0	0,0	500,0	0,0	500,0	0,0	0,0	0,0	0,0
Suriella sp.	0,0	0,0	0,0	800,0	0,0	0,0	0,0	0,0	0,0	0,0
Aulacoseira granulata (Ehrenberg) Simonsen	0,0	623,1	934,7	934,7	623,1	383,2	0,0	0,0	623,1	520,3
Unidentified 1	0,0	0,0	0,0	0,0	0,0	966,6	0,0	0,0	0,0	0,0
Unidentified 2	0,0	406,6	0,0	0,0	0,0	0,0	0,0	0,0	0,0	0,0
Total	**2500,0**	**7429,8**	**7373,9**	**4584,7**	**1100,1**	**3626,8**	**240,1**	**1180,0**	**923,1**	**1460,3**
